# The Effect of Different Coupling Agents on Nano-ZnO Materials Obtained via the Sol–Gel Process

**DOI:** 10.3390/nano7120439

**Published:** 2017-12-12

**Authors:** Violeta Purcar, Raluca Şomoghi, Sabina Georgiana Niţu, Cristian-Andi Nicolae, Elvira Alexandrescu, Ioana Cătălina Gîfu, Augusta Raluca Gabor, Hermine Stroescu, Raluca Ianchiş, Simona Căprărescu, Ludmila Otilia Cinteză

**Affiliations:** 1 R&D National Institute for Chemistry and Petrochemistry-ICECHIM, 202 Spl. Independentei, 6th District, 060021 Bucharest, Romania; purcarvioleta@gmail.com (V.P.); sabina.nitu@icechim-pd.ro (S.G.N.); cristian.nicolae@icechim-pd.ro (C.-A.N.); elvira.alexandrescu@icechim-pd.ro (E.A.); catalina.gifu@icechim-pd.ro (I.C.G.); raluca.gabor@icechim-pd.ro (A.R.G.); raluca.ianchis@icechim-pd.ro (R.I.); 2 Institute of Physical Chemistry “Ilie Murgulescu” of the Romanian Academy, 202 Spl. Independentei, 6th District, 060021 Bucharest, Romania; hstoescu@icf.ro; 3 Faculty of Applied Chemistry and Materials Science, Politehnica University of Bucharest, 1-7 Polizu Str., 1st District, 011061 Bucharest, Romania; s_caprarescu@chim.upb.ro; 4 Physical Chemistry Department, University of Bucharest, 4-12 Elisabeta Blvd., 1st District, 030118 Bucharest, Romania; ocinteza@gw-chimie.math.unibuc.ro

**Keywords:** sol–gel process, ZnO nanoparticles, coupling agent, surface modifications, hybrid coating

## Abstract

Hybrid nanomaterials based on zinc oxide were synthesized via the sol–gel method, using different silane coupling agents: (3-glycidyloxypropyl)trimethoxysilane (GPTMS), phenyltriethoxysilane (PhTES), octyltriethoxysilane (OTES), and octadecyltriethoxysilane (ODTES). Morphological properties and the silane precursor type effect on the particle size were investigated using dynamic light scattering (DLS), environmental scanning electron microscopy (ESEM), transmission electron microscopy (TEM), thermogravimetric analysis (TGA), and X-ray diffraction (XRD). The bonding characteristics of modified ZnO materials were investigated using Fourier transform infrared spectroscopy (FTIR). The final solutions were deposited on metallic substrate (aluminum) in order to realize coatings with various wettability and roughness. The morphological studies, obtained by ESEM and TEM analysis, showed that the sizes of the ZnO nanoparticles are changed as function of silane precursor used in synthesis. The thermal stability of modified ZnO materials showed that the degradation of the alkyl groups takes place in the 300–500 °C range. Water wettability study revealed a contact angle of 142 ± 5° for the surface covered with ZnO material modified with ODTES and showed that the water contact angle increases as the alkyl chain from the silica precursor increases. These modified ZnO materials, therefore, can be easily incorporated in coatings for various applications such as anti-corrosion and anti-icing.

## 1. Introduction

The variety of structures of nanometric zinc oxide can be classified among new materials with potential applications in various branches of industry: rubber, pharmaceutical, cosmetics, textile, electronic and electrotechnology, photocatalysis, and pro-ecological systems [1,2,3]. Various techniques for the preparation of zinc oxide (ZnO) nanopowders, such as sol-gel processing, co-precipitation, vapor deposition, precipitation in water solutions, hydrothermal synthesis, precipitation from microemulsions and mechanochemical processes, mechanical milling, organometallic synthesis, and the microwave method [4,5,6,7,8,9,10,11], have been applied have enabled products with ZnO particles differing in shape, size, and spatial structure. Among the different methods, the sol–gel approach appears to be one of the most promising methods to prepare ZnO nanoparticles due to several advantages: the ease of synthesis, low temperature of decomposition, control over the chemical composition, low cost, reliability, repeatability, and relatively mild synthesis conditions. In recent years, studies have focused on sol–gel formulations that afford not only good barrier properties but also inhibitive characteristics [12,13,14]. Silica-coated ZnO nanomaterials are of particular interest because they have good environmental stability with different materials [15] and surfaces are easy to modify [16,17]. Moreover, silane coupling agents have been selected to modify ZnO nanoparticles because they can inhibit nanoparticle growth and enhance the long-term stability in organic matrices [18]. Some research has shown that the types and amounts of silane coupling agents influence the properties of nanomaterials, such as the morphology, processability, and optical, mechanical, and barrier properties [19,20]. Surface modification of ZnO nanoparticles is a practical means of changing chemical and physical properties and improving dispersion in organic media. For example, triethoxysilane treatments on thin sol–gel grown films of ZnO were reported by C.G. Aleen et al. [21]. It was demonstrated that, by using different R-triethoxysilane precursors (R = octadecyl-, decyl-, phenyl-), it is possible to achieve a broad range of surface functionalizations through variation of the R end group. D. Álvarez et al. [22] proposed the functionalization of zinc oxide nanoparticles using (3-glycidyloxypropyl)trimethoxysilane (GPTMS) to assess the effect of surface modification. The obtained results confirmed that the addition of ZnO nanoparticles to the sol–gel film improves the protection properties of the system. M. Jiang et al. [23] demonstrated that the incorporation of silane agents improves the performance of epoxy coatings on alloy substrates. It was demonstrated that the amine and thiol organofunctional groups are covalently attached to the silica layer. H. Chen et al. [24] prepared hydrophobic ZnO nanoparticles using aminopropyltriethoxysilane (APTES) as a silane coupling agent. It was demonstrated that APTES was linked to the surface of ZnO nanoparticles through chemical bond bindings. Y. N. Li et al. [25] modified commercial zinc oxide nanoparticles using APTES and GPTMS as silane coupling agents. The obtained results suggest that the modifier of silane can change the surface hydrophilicity and effectively break the agglomerations of nanoparticles. Grasset et al. [26] coated commercial ZnO nanoparticles with APTES under varying environments and demonstrated that the ZnO silane coating can be used to obtain a homogeneous dispersion of photostable nanoparticles. G. A. Farzi et al. [27] modified zinc oxide nanoparticles with different mole ratios of trimetoxyvinyl silane and oleic acid, used as coupling agents, to modify their surface properties. It was shown that the combination of these two coupling agents allows for the control of the hydrophobicity and dispersibility of the ZnO nanoparticles in the solvents with varying polarity.

The present study focuses on the preparation of modified zinc oxide (ZnO) materials by the sol–gel process using different silane precursors such as (3-glycidyloxypropyl)trimethoxysilane (GPTMS), phenyltriethoxysilane (PhTES), octyltriethoxysilane (OTES), and octadecyltriethoxysilane (ODTES) as modifying agents. Properties of the modified ZnO materials, such as size distribution, morphology, and structure were characterized through dynamic light scattering (DLS), environmental scanning electron microscopy (ESEM), transmission electron microscopy (TEM), Fourier transform infrared (FTIR) spectroscopy, thermogravimetric (TGA) analysis, and X-ray diffraction (XRD). Since the wettability of solid surfaces is an interesting topic in materials science, due especially to a large area of potential applications (e.g., anti-corrosion and anti-icing), we also investigated the water-repellence behavior of films deposited onto a metallic substrate (aluminum). We carried out the roughness and water contact angle (CA) measurements and correlated the obtained results with the structural and morphological properties.

## 2. Results and Discussion

**Figure 1:**
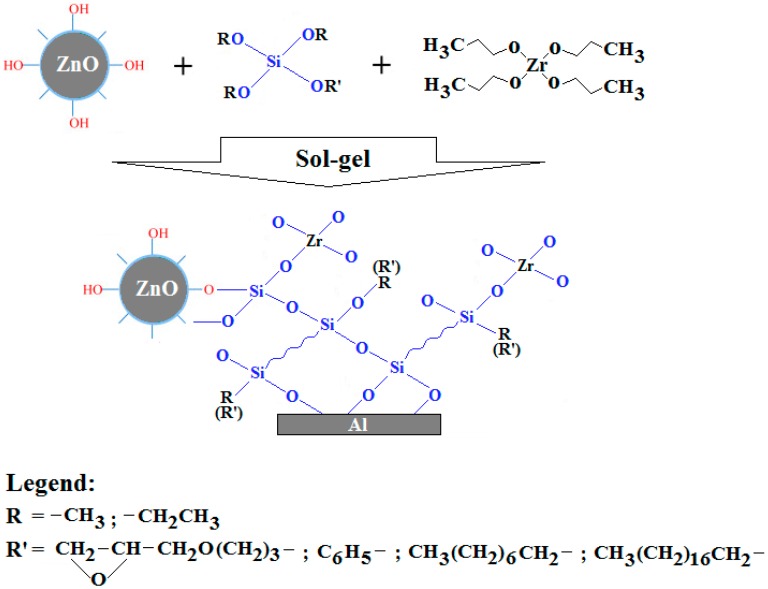
Probable reaction mechanism that can occur when ZnO is modified using silane precursors.

In this paper, nanomaterials based on zinc oxide (ZnO) were synthesized via the sol–gel method, using different silane coupling agents—(3-glycidyloxypropyl)trimethoxysilane (GPTMS), phenyltriethoxysilane (PhTES), octyltriethoxysilane (OTES), and octadecyltriethoxysilane (ODTES), as modified agents—in the presence of a zirconium IV propoxide solution (ZPO) as an inorganic precursor. The resulting modified ZnO materials were characterized both as dispersions and as films deposited onto a metallic substrate (aluminum) (Figure 1).

**Figure 2:**
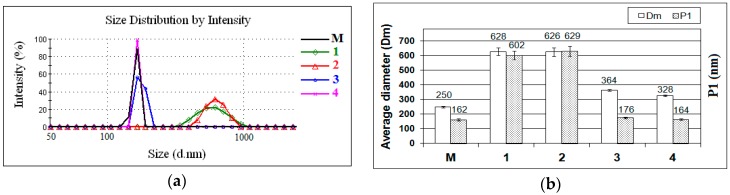
(**a**) Particle size distributions and (**b**) average diameters (Dm) and main peak (P1), obtained for unfunctionalized ZnO (M) and for ZnO nanoparticles modified with (1) GPTMS, (2) PhTES, (3) OTES, and (4) ODTES, respectively.

The particle size distributions of commercial ZnO nanoparticles (M) and of ZnO nanoparticles modified by different silane precursors are shown in Figure 2a. The size variation of all samples (in terms of average diameter (Dm) and main peak (P1)) is shown in Figure 2b. The type and functionality of the silanes played an important role in the experiment. In all samples, only one peak can be observed (monomodal distribution). Different average hydrodynamic diameters were measured. The ZnO nanoparticles (M) present a small size distribution with the average diameter (Dm) about 250 nm. The lower value of the main population of ZnO nanoparticles (characterized by P1, ~162 nm), compared with Dm, can be explained by the presence of smaller particles. The obtained results reveal that the size average diameters of modified ZnO nanoparticles are clearly different depending on the silane precursor. It can be seen that the large particle size distribution (where Dm ~ 630 nm) is obtained when the silane precursor with a short alkyl chain (e.g., glycidyoxy-, phenyl-) is used in the synthesis (Samples 1 and 2). This fact can be explained by the hydrolytic condensation reactions produced between the zinc hydroxide and the silanol groups of the precursor (GPTMS or PhTES). Thus, not all silanol groups can react with ZnO nanoparticles, maybe due to steric effects. It is possible to observe that Samples 3 and 4 present a small particle size distribution (where Dm ~ 350 nm and P1 ~ 170 nm). Steric factors strongly influenced the silica precursors with octyl- or octadecyl- functionalities, resulting in modified ZnO nanoparticles with a small particle size (a longer alkyl chain leads to a lower hydrolysis rate). Although the recorded main peak (P1) of ZnO nanoparticles modified with OTES (Sample 3) and ODTES (Sample 4) indicated, in general, smaller particle sizes than those measured for Samples 1 and 2, values of the average diameters (Dm) were much higher, due to the formation of large aggregates in the hybrid systems. These results confirm that the hydrolysis of silica precursors with a long alkyl chain leads to monomeric units of the corresponding hydroxides that can act as active centers for the polycondensation of an Si–O–Si network.

The morphology and size of unfunctionalized ZnO nanoparticles (M) and of ZnO nanoparticles modified using different silane precursors (Samples 1–4) were examined by environmental scanning electron microscopy (ESEM) and transmission electron microscopy (TEM).

**Figure 3:**
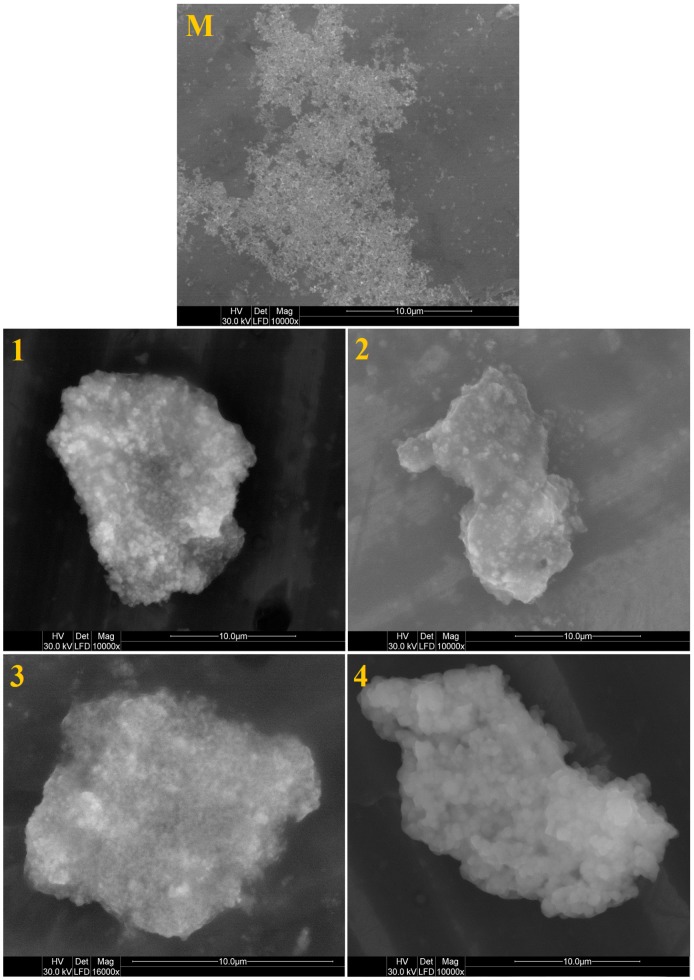
ESEM images of unfunctionalized ZnO nanoparticles (M) and of ZnO nanoparticles modified with (1) GPTMS; (2) PhTES; (3) OTES; and (4) ODTES, respectively.

Figure 3 shows the ESEM images of unfunctionalized ZnO nanoparticles (M) and ZnO nanoparticles modified with different silane precursors (characterized as dispersions, Samples 1–4). The unfunctionalized ZnO nanoparticles (M) present a small size diameter. The ESEM pictures illustrate the different of the effect of silane precursor type on the ZnO nanoparticles. It can be seen from these images that the ZnO nanoparticles are embedded into a sol–gel matrix. ZnO nanoparticles modified with precursors having long alkyl chains (Samples 3 and 4) presents monodisperse particles with an average diameter of about 150 nm, but with a significant decrease in the surrounding matrix than that synthesized with precursors possessing short alkyl chains (Samples 1 and 2); this can be an effect of the ZnO nanoparticle modification with a long organic chain from the silane precursor, which prevents the formation of aggregates due to the steric hindrance between inorganic nanoparticles [28]. These results are in good agreement with DLS measurements, which confirm that the ZnO nanoparticles modified with short alkyl chains present a large particle size distribution. Thus, for Samples 3 and 4, the DLS data refer not to aggregates but to single hydrated particles.

**Figure 4:**
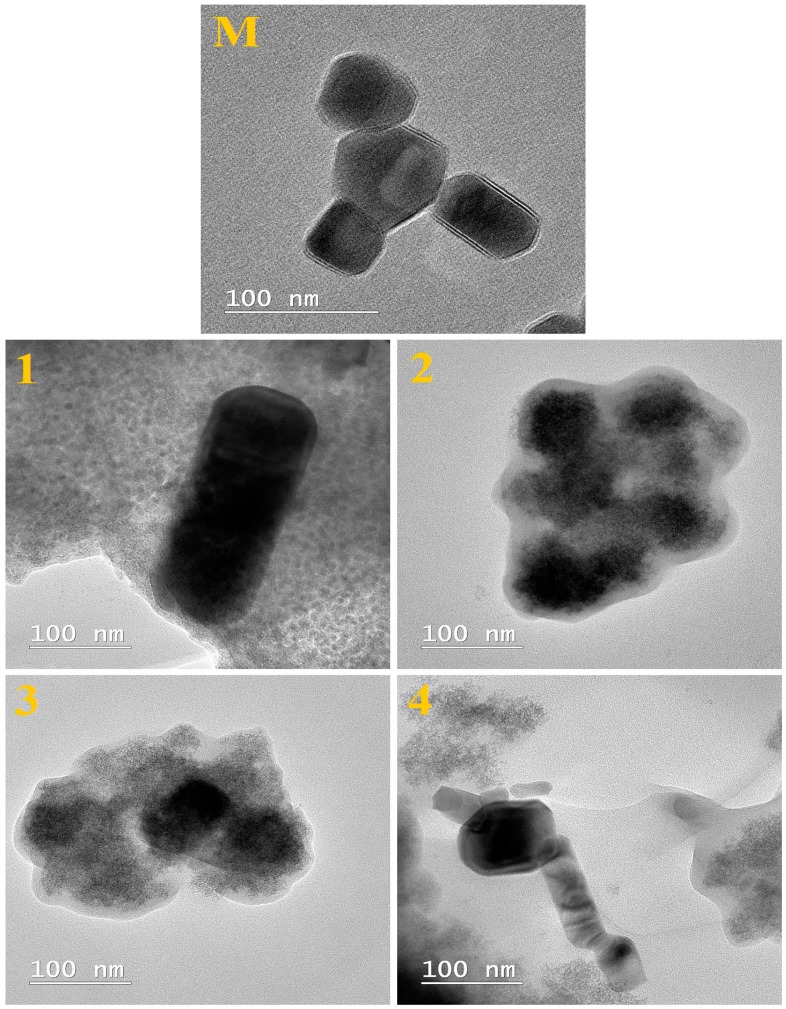
TEM images of unfunctionalized ZnO nanoparticles (M) and of ZnO nanoparticles modified with (1) GPTMS, (2) PhTES, (3) OTES, and (4) ODTES, respectively.

TEM images of ZnO nanoparticles (M) and of modified ZnO materials (Samples 1–4) are given in Figure 4. It can be seen from TEM images that the ZnO nanoparticles are embedded into a sol–gel matrix. The silica nanoparticles were evident as a gray structure with the zinc oxide appearing as dark nanoparticles (dark color), which were probably formed by the condensation process of silica solution in the presence of silane precursor.

**Figure 5:**
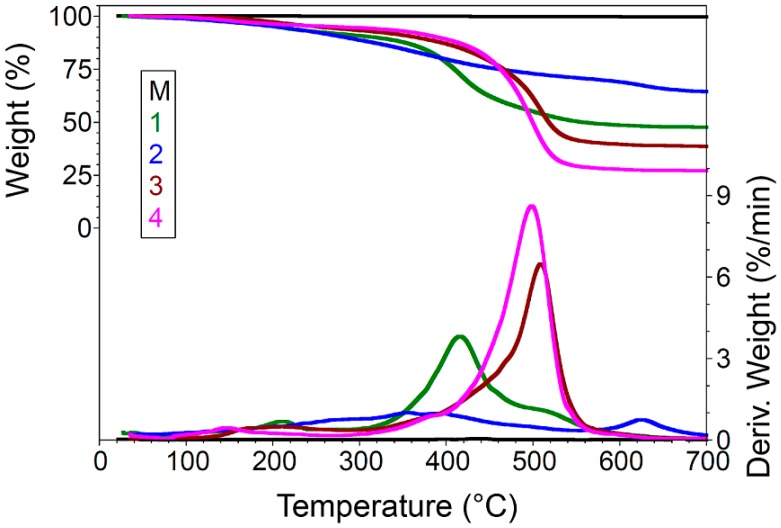
TGA curves of unfunctionalized ZnO (M) and of dried ZnO materials modified with (1) GPTMS, (2) PhTES, (3) OTES, and (4) ODTES,; respectively.

Thermogravimetric analysis (TGA) of unfunctionalized ZnO (M) and of modified ZnO materials was examined under a nitrogen atmosphere at 40–700 °C at 10 °C/min (Figure 5). The TGA curve for the unfunctionalized ZnO (M) shows a very small decrease in weight percentage at around 220 °C. It can be seen from Figure 5 and Table 1 that the modified ZnO materials have a weight loss before 275 °C, which is related to the elimination of physically absorbed water on the surface and residual organics from the process of the ZnO synthesis [29]. Water elimination at higher temperatures is also caused by the hydroxyl pair condensation. Surface silanols are either free Si–OH groups or hydrogen-bonded with water or vicinal silanol groups. The difference between the total losses of the modified ZnO materials is due to the organofunctional group of the silica precursor. The modified ZnO nanoparticles begin to lose weight continuously after 300 °C, which contributed to the debonding and degradation of the attached silane functional group on the surface [30]. The char yields at 700 °C of the ZnO materials modified with the silane precursor with a short alkyl chain (Samples 1 and 2) are higher than that of ZnO materials containing a silane precursor with a long alkyl chain (Sample 3 and 4). This difference in thermal stability may be a result of the dense coverage of thermal silica with long-chained silanes [31].

**Figure 6:**
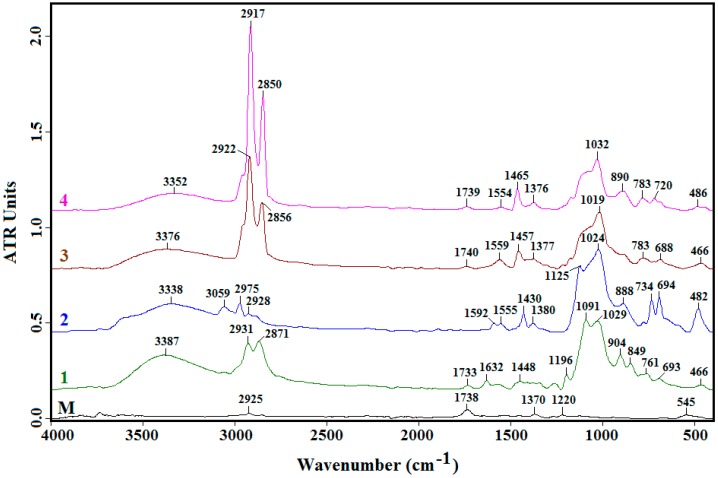
FTIR spectra of unfunctionalized ZnO (M) and of ZnO nanoparticles modified with (1) GPTMS, (2) PhTES, (3) OTES, and (4) ODTES, respectively, deposited onto an aluminum substrate.

Analyzing the FTIR spectra (Figure 6) of unfunctionalized ZnO nanoparticles and of modified ZnO materials, the broad band at around 3390–3350 cm^−1^ is observed and corresponds to the overlapping of the O–H stretching bands of hydrogen-bonded water molecules (H–O–H) and the SiO–H stretching of surface silanols hydrogen bonded to molecular water (SiO–H···H_2_O) [32].

In the FTIR spectrum of the ZnO, a band located at 545 cm^−1^, correlated to the stretching mode of Zn–O, can be observed. All FTIR spectra of modified ZnO materials exhibit a broad absorption band located at ~1030 cm^−1^, which is assigned to the Si–O–Si asymmetrical stretching vibrations and confirms that the alkylsilane molecules are covalently attached to the ZnO. It has been established that the peak frequencies of the C–H stretching modes are a good indicator of the conformation of alkyl chains (–CH_2_ and –CH_3_, symmetrical and asymmetrical stretching) [33]. For Samples 3 and 4, the symmetric (*ν*_s_) and asymmetric (*ν*_a_) peaks were located at ~2920 and ~2850 cm^−1^, respectively. This result is in good agreement with a previous report of alkylsiloxane modification of SiO_2_ substrates [34]. In case of Samples 1 and 2, the *ν*_a_ and *ν*_s_ peaks were shifted toward higher wavenumbers, likely caused by the conformational disorder of alkyl chains.

In the FTIR spectra of ZnO material modified with GPTMS (Sample 1), the peaks at 693 and 904 cm^−1^ are assigned to the Si–O symmetric stretching vibration and to the in-plane stretching vibration of free SiO–, respectively [35]. The peak at 2931 cm^−1^ is attributed to the stretching vibration of the C–H asymmetry from GPTMS [36].

For the hybrid material containing ZnO–phenyl (Sample 2), the peaks that occurred at 694 and 734 cm^−1^, respectively, are attributed to the phenyl H out-of-plane ring deformation vibration. The Si–phenyl peak was observed at 1430 cm^−1^, assigned to the same vibrational combination [37].

In the FTIR spectra of Samples 3 and 4 obtained with OTES and ODTES, respectively, a peak at ~1460 cm^−1^ is revealed and is attributed to a symmetric bending vibration of the CH_3_ group [38]. The peak identified at 786 cm^−1^ is due to Si–O(Si) and Si–O(C) bonds (symmetric stretching vibration) [39].

The presence of the peak at 450–500 cm^−1^ of Si–O–Si confirmed the ZnO–Si bond formation [23,40].

**Figure 7:**
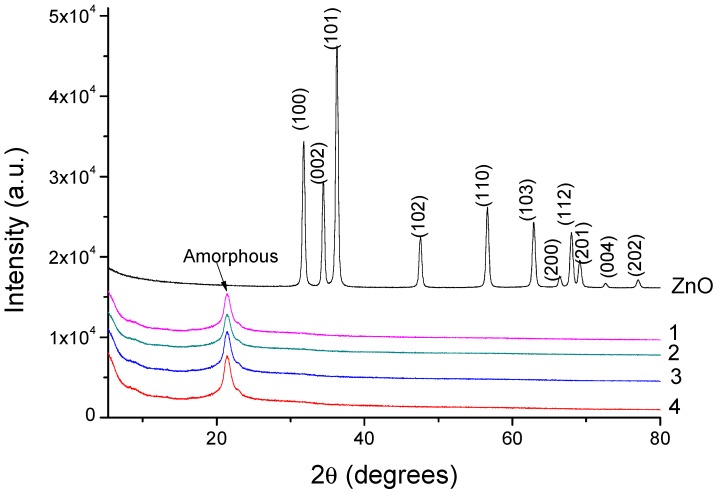
XRD patterns of unfunctionalized ZnO (M) and of dried ZnO materials modified with (1) GPTMS, (2) PhTES, (3) OTES, and (4) ODTES, respectively.

Figure 7 presents the XRD patterns of unfunctionalized ZnO (M) and of dried ZnO materials modified with silane precursors. The unfunctionalized ZnO (M) present a crystalline structure, as demonstrated by peaks in the XRD scan associated with the (100), (002), (101), (102), (110), (103), and (112) crystal planes. The dried ZnO materials modified with silane precursors showed no XRD peaks, because silica precursors are, in general, non-crystalline. Another reason is the very slow rate of condensation reactions that decline the formation of Zn–O clusters in the sol and hinder the formation of crystalline structure.

The wetting ability of coatings was evaluated by contact angle (CA) measurements. The CA is a measure of adhesion between liquid droplet (water) and solid surface. One purpose of ZnO modified with silane precursor is to obtain silane functional groups that can change the ZnO material surface from a hydrophilic nature to a hydrophobic nature [17]. The reason that the long alkyl group of silane is superior to the short alkyl group of silane in terms of nanosilica modification is related to the hydrophobicity of their functional groups.

**Figure 8:**
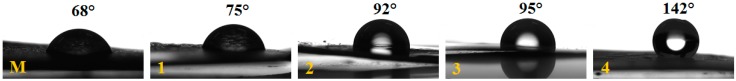
Profiles of water droplets deposited onto metallic substrate, covered with unfunctionalized ZnO (M) and with ZnO nanoparticles modified with (1) GPTMS, (2) PhTES, (3) OTES, and (4) ODTES, respectively.

Figure 8 shows the profiles of water droplets deposited onto the metallic substrate, covered with unfunctionalized ZnO (M) and with ZnO nanoparticles modified with different silane precursors. The water CA obtained from the coating with unfunctionalized ZnO nanoparticles was 68 ± 3°. The lower CA recorded from the ZnO sample modified with GPTMS (75 ± 3°), compared to those treated with other silica precursors, was partially caused by conformational disorder of the epoxy alkyl chain [21,41]. This indicates that the Si–O–Si bonds are not stable when the GPTMS is used as a silane precursor. In this case, reactions of Si–O–Si can suffer hydrolysis, forming the Si–OH hydrophilic groups and allowing water droplets to penetrate the surface [42].

The hydrophobic character of the modified ZnO materials, deposited on the aluminum substrate, increases as the hydrophobic chain length increases, in the following order: PhTES < OTES < ODTES. The hydrophobic Si–R groups (where R = phenyl, octyl, octadecyl) lead to improved hydrophobic properties in the layer coatings. The hydrolytically stable functional Si–R groups and the ZnO particle form a protective layer on the coating’s surface as a result of the silylating reactions between alkoxy groups and Zn atoms on the surface, which prevents the adsorption of water [43]. Modification of ZnO nanoparticles with ODTES allows an ideal micro/nano rough topography, as evident from Figure 8 (Sample 4). The existence of this micro/nano structure leads to the porous structure of the ZnO coating, facilitating the formation of an ultra-hydrophobic surface with a water contact angle of 142 ± 5° [44,45]. For Samples 2 and 3, the degree of roughness was not sufficient, and ultra-hydrophobicity was not achieved. The CAs recorded for these samples were 92 ± 4° and 95 ± 6°, respectively, indicating that the functional hydrophobic groups started to adhere to the metallic surface.

Table 2 presents the roughness results for the investigated samples. These results show a tendency of increasing sample roughness in the series, from the unfunctionalized ZnO (M) toward Sample 4, with the exception of Sample 2, which had a greater increase in roughness.

These results are consistent with the conclusion that long organic groups from a silane precursor promote a higher hydrophobicity of the surfaces.

## 3. Experimental Section

### 3.1. Materials

Commercial ZnO nanopowder (<100 nm, ZnO, Sigma-Aldrich, Philadelphia, PA, USA), (3-glycidyloxypropyl)trimethoxysilane (GPTMS, 97%, Fluka, Philadelphia, PA, USA), phenyltriethoxysilane (PhTES, 98%, Fluka, Philadelphia, PA, USA), octyltriethoxysilane (OTES, 97.5%, Sigma-Aldrich, Philadelphia, PA, USA), octadecyltriethoxysilane (ODTES, 98%, Alfa Aesar, Karlsruhe, Germany), nitric acid (HNO_3_ 100%, Merck, Darmstadt, Germany), ethyl-acetoacetate (99%, Bucharest Reactive, Bucharest, Romania), 2-propanol (99.9%, SC CHIMREACHIV SRL, Bucharest, Romania) were used as purchased. Zirconium IV propoxide solution (ZPO, 70 wt % in 1-propanol, Sigma-Aldrich, Philadelphia, PA, USA) was used as the inorganic precursor. A metallic substrate (99.99% aluminum, Shanghai, China); surface roughness: 5.87 nm, mean squared error (MSE: 0.43) was used to obtain coatings. The metallic material was cut into 1 × 1 cm^2^ coupons.

### 3.2. Synthesis of Modified ZnO-Nanoparticles

The modified ZnO materials were prepared through a sol–gel process. Briefly, in order to prepare the inorganic sol, 1.2 mL of zirconium IV propoxide solution (ZPO) was added dropwise in 1.2 mL of ethyl-acetoacetate, mixed together, and magnetically stirred for 20 min and ultrasonically stirred for another 20 min at 40 °C. Then, 0.25 mL of HNO_3_ solution (pH = 0.5) was added to the solution and ultrasonically stirred for 100 min. Meanwhile, the organic sol was prepared by mixing 2.5 mL of 2-propanol and silane precursor (1.1 × 10^−2^ moles GPTMS, PhTES, OTES, or ODTES) with 0.325 mL of HNO_3_ solution (pH = 0.5) and magnetically stirred for 60 min at 40 °C. Finally, both sols were mixed together for another 60 min, keeping the same temperature. To obtain modified ZnO nanoparticles, 1 mL of ZnO nanoparticles dispersed in 2-propanol (a concentration of 0.5% (*w*/*w*)) was mixed with 1 mL of final sols and ultrasonically stirred for 10 min. The resulted solutions were left at rest for 24 h and then characterized both as dispersions and as films deposited onto the metallic substrate (aluminum) (see Figure 1).

The metallic substrate, to ensure uniform wetting, was cleaned with soap, distilled water, and finally with ethanol. The sol–gel films were obtained by dipping the metallic substrate in final solutions. All final samples deposited onto metallic substrate were dried and kept (overnight) at room temperature (25 °C).

### 3.3. Characterization Methods

Particle size distribution was determinate by dynamic light scattering (DLS) (ZetasizerNanoZS instrument Malvern Instruments Ltd., Malvern, UK). The determinations for samples (0.1 mL samples diluted in 25 mL of 2-propanol and ultrasonicated for 5 min) were performed at 25 °C. Morphology and shape of the resulting modified silica ZnO particles (0.1 mL samples diluted in 25 mL of ethanol and ultrasonicated for 5 min) were studied via environmental scanning electron microscopy (ESEM) using an FEI-Quanta 200 microscope and via transmission electron microscopy (TEM) with a Tecnai™ G2 F20 TWIN Cryo-TEM instrument (FEI Company, Eindhoven, The Netherlands) at 200 kV acceleration voltages. ESEM images were obtained in a low vacuum mode, without any covering of the samples. In the case of TEM analysis, the samples were observed directly without further staining for improving contrast. A droplet of diluted sample was poured on a carbon film-coated copper grid and left to air-dry at room temperature. Thermal analysis of the modified ZnO materials (dried under vacuum at 50 °C for 24 h) was performed using a Thermogravimetric Analyser TGA Q5000IR (TA Instruments, New Castle, DE, USA) in a nitrogen atmosphere (10 °C/min) at 40–700 °C. The internal structure’s modifications of the dried modified ZnO materials deposited on a metallic substrate was subjected to Fourier transform infrared spectroscopy using a Spectrometer Tensor 37 (Bruker Instrument, Woodstock, NY, USA), in ATR mode with a Golden Gate diamond unite (400–4000 cm^−1^), resolution of 4 cm^−1^. X-ray diffraction (XRD) analyses were achieved by the use of a SmartLab diffractometer (RigakuEurope SE, Ettlingen, Germany). The operating conditions were as follows: 45 kV, 200 mA, Cu Kα radiation (1.54059 Å), and a parallel beam configuration (2θ/θ scan mode), from 5 to 90° 2θ. The static contact angle (CA) of deionized water on deposited films was measured through the Drop Shape Analysis System, model DSA1 (FM40 Easy Drop, DIP-ROBOT DR-3, Riegler and Kirstein, Berlin, Germany), at room temperature in air. The sample was placed on a plane stage using a stainless steel needle with an outer diameter of 0.5 mm. All CA measurements were carried out in a static regime at room temperature with a drop volume of 3 µL. Contact angles were obtained by fitting the drop shape with a mathematical expression. Then, the slope of the tangent to the drop at the liquid–solid–vapor (LSV) interface was calculated. The reported contact angle values were obtained as the average of five measurements (liquid droplets placed in various regions of the film surface). The spectroscopic ellipsometry (SE) measurements were carried out at room temperature on a variable angle spectroscopic ellipsometer (VASE, J.A. Woollam. Co., Lincoln, NE, USA) in a spectral range of 400–1000 nm at a 70° angle of incidence. For the ellipsometric data simulation, the Cauchy model was applied in the transparent region, and surface roughness was obtained.

## 4. Conclusions

Modified ZnO materials were prepared through the sol–gel process using different silica precursors—(3-glycidyloxypropyl)trimethoxysilane (GPTMS), phenyltriethoxysilane (PhTES), octyltriethoxysilane (OTES), and octadecyltriethoxysilane (ODTES)—and were characterized via DLS, ESEM, TEM, TGA, FTIR, and roughness and water contact angle analysis. DLS characterization revealed a dependence of the particle’s size on the type of substituting organic groups from silica precursors. The ZnO particles were dispersed into the silica matrix, confirmed by ESEM and TEM. The thermal stability of the dried ZnO materials indicated that silica nanoparticles began to lose hydrophobicity when they were heated in the 300–500 °C range. FTIR spectra revealed that the organofunctional groups were attached to the silica surface. The modified ZnO materials had an amorphous character, as shown by XRD analysis. Contact angle (CA) measurements showed that the ZnO silica surface can be changed from a hydrophilic nature to a hydrophobic nature using a silane precursor with a long alkyl chain. Coating with modified ZnO material containing ODTES presents a higher degree of nanometer-scale roughness (CA = 142 ± 5°). These obtained ZnO materials can be used for anti-icing and corrosion protection of metallic structures.

## Figures and Tables

**Table 1:** Thermal degradation of unfunctionalized ZnO (M) and of dried ZnO materials modified with different silane precursors.

Sample No.	40–275 °C		275–575 °C				575–700 °C		Residue at 700 °C
	Wt. Loss %	*T*_max1_^1^ °C	Wt. Loss %	*T*_max2_ °C	*T*_max3_ °C	*T*_max4_ °C	Wt. Loss %	*T*_max5_ °C	N_2_
M	0.11	216.80	0.22	-	436.90	-	0.02	-	99.65
1	8.40	212.00	42.46	358.20	415.80	518.60	1.70	624.60	47.41
2	9.51	270.20	20.60	356.60	390.90	492.80	5.68	623.50	64.22
3	6.02	211.40	53.91	369.90	429.20	508.80	1.65	600.50	38.42
4	5.18	146.40	66.70	379.20	417.10	498.20	1.23	609.60	26.88

^1^ *T*_max_ (°C) = *T*(*d*α/*d*t)_max_.

**Table 2:** Roughness and MSE (mean squared error) of coatings with ZnO materials modified with different silane precursors.

Sample No.	Sample Roughness (nm)	MSE
M	2.69	0.55
1	7.35	1.43
2	27.06	1.18
3	13.31	1.21
4	54.13	1.29
